# Imaging biomarkers associated with extra-axial intracranial tumors: a systematic review

**DOI:** 10.3389/fonc.2023.1131013

**Published:** 2023-04-25

**Authors:** Navodini Wijethilake, Oscar MacCormac, Tom Vercauteren, Jonathan Shapey

**Affiliations:** ^1^ School of Biomedical Engineering and Imaging Sciences, King’s College London, London, United Kingdom; ^2^ Department of Neurosurgery, King’s College Hospital NHS Foundation Trust, London, United Kingdom

**Keywords:** extra-axial, intracranial, biomarker, marker, imaging, growth, tumor neoplasms

## Abstract

Extra-axial brain tumors are extra-cerebral tumors and are usually benign. The choice of treatment for extra-axial tumors is often dependent on the growth of the tumor, and imaging plays a significant role in monitoring growth and clinical decision-making. This motivates the investigation of imaging biomarkers for these tumors that may be incorporated into clinical workflows to inform treatment decisions. The databases from Pubmed, Web of Science, Embase, and Medline were searched from 1 January 2000 to 7 March 2022, to systematically identify relevant publications in this area. All studies that used an imaging tool and found an association with a growth-related factor, including molecular markers, grade, survival, growth/progression, recurrence, and treatment outcomes, were included in this review. We included 42 studies, comprising 22 studies (50%) of patients with meningioma; 17 studies (38.6%) of patients with pituitary tumors; three studies (6.8%) of patients with vestibular schwannomas; and two studies (4.5%) of patients with solitary fibrous tumors. The included studies were explicitly and narratively analyzed according to tumor type and imaging tool. The risk of bias and concerns regarding applicability were assessed using QUADAS-2. Most studies (41/44) used statistics-based analysis methods, and a small number of studies (3/44) used machine learning. Our review highlights an opportunity for future work to focus on machine learning-based deep feature identification as biomarkers, combining various feature classes such as size, shape, and intensity.

**Systematic Review Registration:** PROSPERO, CRD42022306922

## 1 Introduction

Extra-axial brain tumors occur at anatomical sites external to the brain parenchyma and account for approximately half of all adult intracranial neoplasms (1). The main anatomical locations from which these tumors most commonly arise include the supratentorial dural region, cerebellopontine angle (CPA) region, sellar and suprasellar regions, pineal region, and intraventricular region (2).

Neoplasms identified as extra-axial brain tumors include meningiomas, metastases, vestibular schwannomas, solitary fibrous tumors, and pituitary tumors. Meningiomas are the most common supratentorial dural-based masses and most frequently arise from the meninges overlying the cerebral convexities. Dural-based metastases from other primary malignancies can also occur, although they are much rarer. Vestibular schwannomas (VS) are the most common tumor type found within the CPA. Meningiomas and metastases also develop less frequently in the CPA region. Pituitary adenoma is the most common tumor found in the sellar region, and macroadenomas often extend into the suprasellar region. Meningiomas are also found in the sellar region, originating from the tuberculum sellae, although these are much less common (2). Out of all primary brain and other central nervous system (CNS) tumors, 39.2% arise from the meninges, while 18.1% arise from the pituitary and craniopharyngeal ducts (1). Thus, extra-axial tumors comprise over half of all brain and CNS tumors in the USA, and behaviorally, most extra-axial tumors are non-malignant (1).

Meningioma is the most common extra-axial intracranial neoplasm, and 81.2% of meningiomas are located in the cerebral meninges. Meningiomas are most common found in children aged 0–14 years, and incidence increases with age. This tumor type is most common among adults over 65. Furthermore, meningiomas are also more common in females compared to males and are thought to arise from the arachnoid cap cells in the arachnoid layer of the meninges (1). In the 5th edition of the WHO CNS tumor classification, meningiomas are grouped into three main grade categories (WHO grades 1–3) that involve 15 different histological subtypes (3). However, a wide range of histological patterns can be seen in meningiomas, and some exhibit mixed patterns. WHO grade 1 tumors are generally slow-growing, whereas grade 2 meningiomas typically demonstrate a higher rate of growth and recurrence following resection (4). WHO Grade 3 meningiomas are the most aggressive, accounting for about 1.2% of meningiomas in the US (5).

Pituitary region tumors are the second most commonly reported brain and CNS tumor histology, with an incidence of 4.36 per 100,000 people. These tumors are also more frequently reported in females than in males. Neoplasms located in pituitary and craniopharyngeal ducts are the most common tumor among children and adolescents (age 0–19 years) (1). Pituitary tumors are not categorized into the WHO grading system; however, the WHO has classified pituitary tumors (most of which are pituitary adenomas) into subtypes based on the immunohistochemistry of pituitary hormones and other molecular and pathological markers. The transcription factors PIT-1, T-PIT, and SF-1 that are involved in the development of pituitary tumors are closely assessed for their characterizations (6). Importantly, these subtypes do not characterize the invasion, recurrence, or aggressiveness of adenomas. Nevertheless, the tumor size and its invasion into the cavernous sinus demonstrated on imaging are considered indicators of recurrence and aggressiveness. In addition, other subtypes that have been shown to be more aggressive (known as high-risk adenomas) include sparsely granulated somatotroph adenomas (growth hormone-releasing tumors) and lactotroph adenomas (prolactin-releasing tumors) in males (7).

Nerve sheath tumors are the third most common non-malignant brain and CNS tumors, of which 75% occur in the CPA (1). VSs arise from Schwann cells in the vestibulocochlear nerve and have unpredictable clinical behavior (8). Approximately 95% of VSs are sporadic unilateral tumors. Bilateral tumors are typically caused by a neurofibromatosis type 2 (NF2) genetic alteration (9). However, the NF2 mutations can also cause increased growth patterns in the sporadic VSs and can be considered a marker of VS tumor growth (10).

Solitary fibrous tumor/hemangiopericytoma (SFT/HPC) are rare intracranial extra-axial tumor types. These two types have different origins and prognoses, with the SFT phenotype having benign behavior while the HPC phenotype having a higher recurrence rate and malignant behavior (11). However, the fifth edition of the WHO Classification of Tumors of the Central Nervous System (CNS) introduces a single term (‘solitary fibrous tumor’) for both, rather than SFT/HPC, and a three-class CNS grading scheme based on histological phenotype and mitotic activity (3). Classic SFT phenotypes are considered WHO grade 1, and HPC phenotypes are considered grades 2 and 3 (12).

A biomarker is an indicator that can be either qualitative or quantitative and can depict an underlying biological process, a disease condition, the severity of the condition, or a response to a therapeutic intervention (13). Traditionally, biomarkers are obtained using molecular-level analysis of the disease. However, in the past couple of decades, advancements in medical imaging have enabled the obtainment of anatomic, functional, metabolic, and physiological measurements that can reflect such molecular substrates of diseases. These measurements are called imaging biomarkers—the features or characteristics that can be determined using medical images such as magnetic resonance imaging (MRI), diffusion-weighted imaging (DWI), perfusion-weighted imaging (PWI), positron emission tomography (PET), etc. (14).

Recently, there has been a growing interest in identifying imaging biomarkers related to oncology due to the rising emphasis on personalized cancer management, also called *precision cancer medicine* (90 14). Imaging is used widely, from tumor detection to staging, monitoring therapy, surgical planning, and surveillance. Imaging biomarkers can therefore play a pivotal role in optimizing patient management and outcomes. The non-invasive behavior of imaging biomarkers has a great potential to provide a comprehensive measurement over the other invasive biomarkers, which only reflect a fragment of a spatially or temporally heterogeneous tumor. Systematic reviews had been conducted to explore the imaging biomarkers of various brain tumors, including gliomas and neuro-oncology (15, 16). But to the best of our knowledge, this is the first systematic review to focus on intracranial extra-axial brain tumors.

This review guides the design of future studies looking at imaging features or biomarkers that may be used as tools for developing personalized treatments for extra-axial brain tumors. Early medical imaging research used basic statistical analysis to investigate associations with tumor prognostic factors. Laterally, interest has moved towards using machine learning and deep learning algorithms for tumor segmentation and prognosis analysis (17, 18). This motivated us to look at the imaging and analysis techniques used to evaluate extra-axial tumors and how this work has evolved over time to incorporate methodological advancements.

In this review, we summarize the imaging biomarkers associated with the growth or poor prognosis of intracranial extra-axial neoplasms.

## 2 Methods

The Preferred Reporting Items for Systematic Reviews and Meta-Analyses (PRISMA) Statement and 2020 updated guidance were used for the preparation of this manuscript (19). The study was registered on PROSPERO, an international prospective register of systematic reviews (CRD42022306922)^1^.

### 2.1 Search strategy

A structured search was performed on the Pubmed, Web of Science, Embase, and Medline databases and included studies from 1 January 2000 to 7 March 2022. The following boolean search criteria were applied:

1. (‘dural-based mass’ OR ‘extra-axial brain tumor’ OR meningiomas OR ‘brain metastasis’ OR neurofibroma OR ‘peripheral nerve sheath tumors’ OR schwannoma OR ‘solitary fibrous tumor’ OR ‘hemangiopericytoma’ OR epidermoid OR ‘pituitary adenoma’ OR ‘pituitary macroadenoma’ OR ‘pituitary microadenoma’ OR ‘pituitary tumor’) AND
2. (imaging OR radiomics) AND
3. (biomarker OR marker) AND
4. (growth OR prognosis OR risk)

### 2.2 Study selection

The articles included in this systematic review were written in English and were peer-reviewed. The eligibility criteria included:

1. the study must not be a case study or a review; and
2. an imaging technique was utilized; and
3. all the subjects used in the study had extra-axial tumors; and
4. the study used imaging feature(s); and
5. the study has assessed the association with growth or growth-related factor.

Full articles were obtained by the first author (NW) and further assessed for eligibility by two independent reviewers (NW and OM). Any discrepancy was resolved through mutual review with the senior author (JS). Covidence was used as a supporting tool throughout the filtering process^2^.

**Figure 1:**
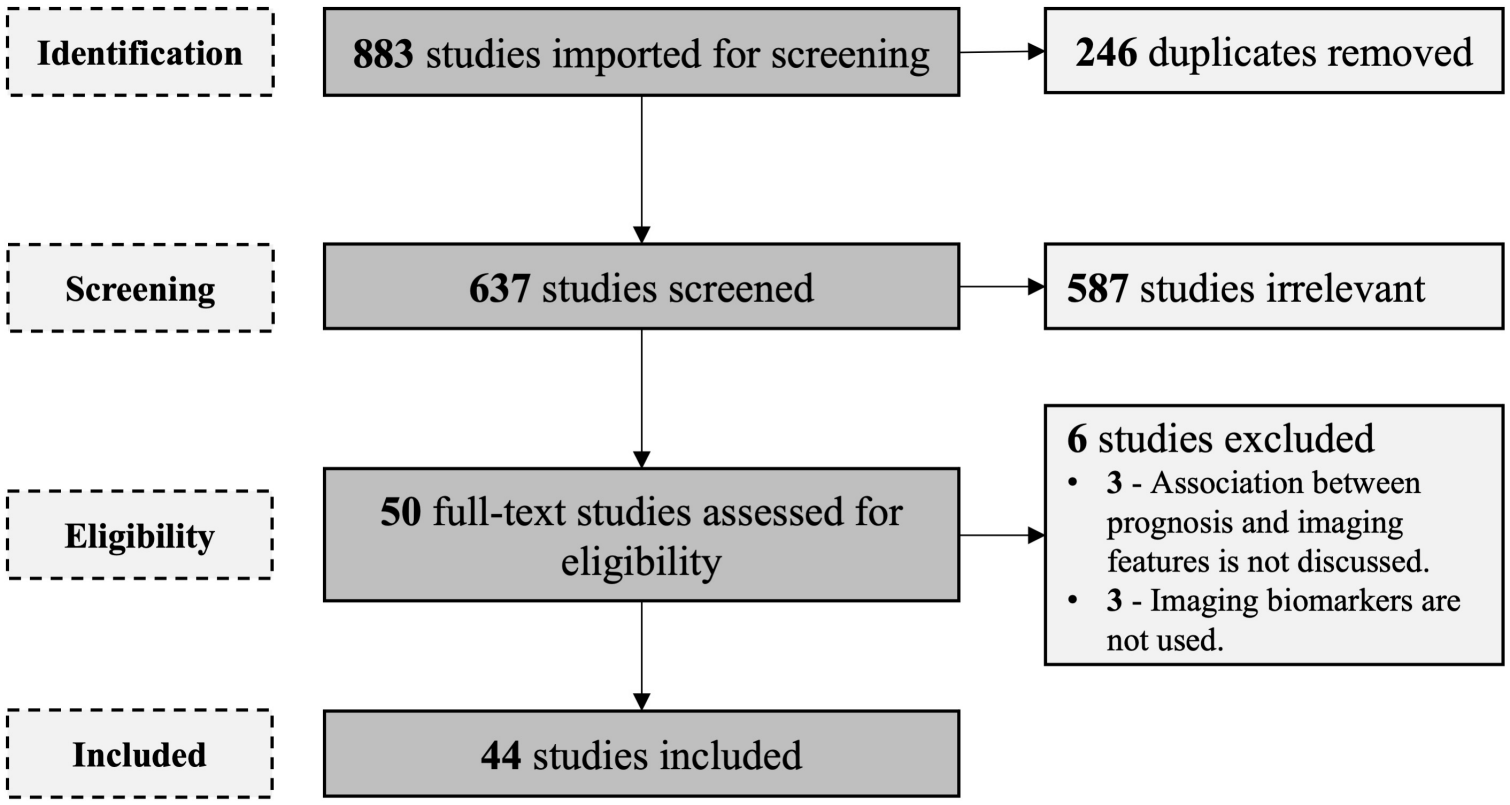
PRISMA flow diagram for the article selection.

In total, 811 studies were filtered by searching databases. After the removal of duplicates, 589 studies were screened by going through the titles and abstracts. This was followed by full-text screening of 49 studies. Six studies were excluded after applying the eligibility criteria. A total of 43 studies satisfied the inclusion criteria and were included in the descriptive analysis 1 (**Figure 1**).

In addition, we defined the outcomes the study should analyze. Since our main aim of the study was to identify growth-related imaging biomarkers, we defined the outcomes we included at the eligibility stage. Studies that assessed imaging biomarkers related to growth related molecular, histopathological, and other markers were considered. Moreover, studies with tumor size monitored before and after treatment, where other pre-treatment imaging biomarkers were assessed, were included. Studies with outcomes not related to growth were excluded.

### 2.3 Data extraction

The included studies were descriptively analyzed based on two main, predefined categories:

1. type of neoplasm,
2. imaging tool used.

In the *Results* section, we discuss our observations in detail.

### 2.4 Quality assessment

The Quality Assessment of Diagnostic Accuracy Studies-2 tool (QUADAS-2) was used to assess the methodological quality of all included studies (20). A quality assessment was performed by the first author (NW). The risk of bias and applicability concerns were assessed. The risk of bias assessment was performed using four QUADAS-2 criteria: i) patient selection; ii) index test; iii) reference standard; and iv) flow and timing. All criteria were scored as ‘low risk,’ ‘high risk,’ or ‘unclear.’ Studies that failed to comment on the criteria or partially commented on the criteria were considered ‘unclear.’

• For patient selection criteria to be ‘low’ risk, patient samples should have been consecutively or randomly selected, and inappropriate exclusions should have been avoided; otherwise, studies were considered ‘high’ risk.
• For the index test, we considered imaging biomarker extraction. If feature extraction was performed blinded to the reference standard, the index test was assessed as ‘low’ risk. If not blinded, the risk of bias is considered ‘high.’
• For the reference standard, we considered outcome-related measurements. In our study, this included histopathological details such as tumor grade and mitotic index. We assessed if this reference standard was acquired while blinded to the index test. Studies fulfilling the criteria were assigned a ‘low’ risk, while those that did not fulfill the criteria were assigned a ‘high’ risk.
• For the flow and timing criteria, we assessed if all the patients who went through the index test received the reference standard and whether they received the same reference standard. Studies fulfilling the criteria were assigned a ‘low’ risk, while those that did not fulfill the criteria were assigned a ‘high’ risk.

Study applicability was assessed on three criteria: (i) patient selection, (ii) index test, and (iii) reference standard. The studies were assigned ‘low,’ ‘high,’ or ‘unclear’ based on the conduct or interpretation of each criteria related to the review question we addressed (can be related, not related, or unclear).

## 3 Results

The imaging biomarkers of the included studies were extracted using four main imaging tools: conventional MRI, DWI, PWI, and PET. Further, the included studies have conducted the corresponding analysis on three main tumor neoplasms: meningiomas, pituitary tumors, and VSs, as we identified after the data extraction. In this section, we explicitly describe the included studies in relation to the two aspects mentioned above. **Table 1** summarizes the included studies.

**Table 1:** Summary of the included studies.

Study	Imaging Tool					Feature Class				Tool			Associated clinical features					
	Conventional MRI	DWI	PWI (DCE-MRI, DSC-MRI)	PET	Other	Intensity/first order statistics	Heterogeneity and texture	Size, Shape, location and Volume	Peritumoral radiomics	Statistical Analysis	Learning Model		Histopathology/Molecular markers	Grade/agressiveness	Survival/Prognosis/Growth	Recurrence	Treatment outcome	Other
Meningiomas																		
Takeda et al. (21)					×	×				×		×						
Ginat et al. (22)			×			×				×		×						
Tang et al. (23)		×				×				×		×						
Seystahl et al. (24)				×		×				×				×	×			
Shi et al. (25)			×			×				×		×						
Gihr et al. (26)		×				×	×			×		×		×				
Gihr et al. (27)	×					×	×			×				×				
Keil et al. (28)			×			×				×		×						
Bashir et al. (29)				×		×				×					×			
Chen et al. (30)	×						×				×			×				
Loewenstern et al. (31)	×							×	×	×					×			
Lu et al. (32)		×				×				×		×						
Bashir et al. (33)				×		×				×		×						
Bashir et al. (34)				×		×				×		×						
Hess et al. (35)	×					×		×		×		×						
Park et al. (36)	×							×		×				×				
Sun et al. (37)	×							×		×				×				
Yu et al. (38)	×	×				×		×	×	×				×				
Bozdağ et al. (39)		×				×			×	×		×		×				
Buizza et al. (40)		×				×				×				×			×	
Feraco et al. (41)																		
Gill et al. (42)	×								×	×					×			
Pituitary tumors																		
Pan et al. (43)	×							×		×		×						
Mahmoud et al. (44)	×	×				×				×		×						
Zhang et al. (45)	×					×		×		×								×
Heck et al. (46)	×					×		×		×				×				
Ceccato et al. (47)	–	–	–	–	–			×		×				×				
Tamrazi et al. (48)		×				×				×								×
Alhambra-Expósito et al. (49)	×					×				×								×
Galm et al. (50)	×					×				×					×	×		
Park et al. (51)	×							×		×		×						
Fan et al. (52)	×					×	×				×						×	
Hasanov et al. (53)	×							×		×		×						×
Ugga et al. (54)	×					×	×			×		×						
Conficoni et al. (55)	×	×				×		×		×		×						
Park et al. (56)	×					×	×			×								×
Swanson et al. (57)	×					×		×		×								×
Lewis et al. (58)	×					×				×						×		
Zhang et al. (59)	×					×	×	×			×					×		
Vestibular schwannomas																		
de Vries et al. (60)	×							×		×		×						
Lewis et al. (61)			×	×		×				×					×			
Lewis et al. (62)			×			×				×					×			
Solitary fibrous tumor																		
Mama et al. (63)	×	×				×				×				×				
Li et al. (64)			×			×				×					×			

### 3.1 Imaging tools used in neuro-oncology

In this section, we discuss the imaging tools used in the studies we included. Conventional MRI was often used, as it is also used routinely in the clinical workflow of extra-axial brain tumor management. Additionally, other tools such as DWI, PWI, and PET were used in the studies we included.

#### 3.1.1 Conventional MRI

MRI is considered the workhorse of brain tumor imaging. MRI can provide macro-structural anatomical information for basic diagnosis and screening of tumors and is routinely used for conventional MRI sequences: T1-weighted MRI (T1) and T2-weighted MRI (T2). Spin echo, fast-field echo, and turbo spin echo are the main techniques used to acquire the above sequences (65). Fluid-attenuated inversion recovery (FLAIR) is the third most commonly used sequence, an inversion recovery sequence with a long inversion time.

T1-weighted MRI may also be acquired after gadolinium contrast agent injection. Contrast-enhanced MRI depicts certain attributes related to the pathophysiology of the tumor by enhancing morphological details within the tumor and also provides basic indications of response to therapy treatments (66). However, contrast agents also carry certain (albeit small) risks associated with patient safety and are also costly compared to imaging without contrast agents.

This imaging tool does not expose the patient to ionizing radiation, posing a low risk. Due to its high sensitivity, MRI is frequently used in brain tumor diagnosis and assessment. In particular, FLAIR is used to detect tumor infiltration beyond the limits of the identified mass (66).

Some advanced MRI techniques, such as DWI and PWI, provide precise, visually differentiable information on microstructural, biophysical, and cellular processes that are also quantitative compared to conventional MRI sequences.

As mentioned above, the routine usage of MRI in clinical workflow is the key motivation behind using conventional MRI in most of the included studies (30). Conventional MRI is more feasible than other advanced imaging techniques (36), and the clear tissue differentiation seen with conventional MRI can provide a region of interest for feature extraction from other co-registered sequences such as DWI (55).

#### 3.1.2 Diffusion-weighted imaging

DWI is extensively used to provide insight into the microscopic tissue structure in neuro-oncology using qualitative and quantitative measures. DWI measures the Brownian motion of water molecules between the intracellular and extracellullar spaces, as well as within the extracellular space. Thus, it is sensitive to fine physiological changes that occur in the tissues (67). DWI does not require the administration of contrast agents and utilizes the conventional spin-echo T2 imaging sequence, in which two additional gradient pulses are applied. When water molecules are in low motion, DWI generates a high signal; this is known as a restriction. The parameter controlling the diffusion sensitivity of DWI, known as the “b value,” depends on the gradient amplitude, duration of the applied gradient, and time gap between two gradients. DWI is useful in tumor detection as it can differentiate tumors as they are more cellular than normal tissue, causing diffusion reduction/impairment (68).

The apparent diffusion coefficient (ADC) map provides a measure of the diffusion magnitude from the DWI by eliminating T2 weighting. ADC maps are frequently used as a visual, qualitative measure. In addition, ADC values can be extracted for specific regions of interest from the ADC map as a quantitative measure. The microstructural information about cellular density is reflected in the ADC measurements and has proven to be useful for histologic differentiation of meningiomas over conventional MRI (69). This has been a key reason for using ADC maps for analyzing meningioma-related grading and histopathologies in several included studies (23, 26, 32, 39).

Diffusion anisotropy is unequal directional diffusion that occurs due to the organization of cells and tissues and can be assessed using DWI. This measurement helps clinicians identify the invasion of the tumor to adjacent structures (e.g., white matter tracts) and the malignancy of the tumor as the heterogeneity within the tumor causes the diffusion to become isotropic (70, 71).

However, DWI has limitations, including a lack of standardization in assessing and analyzing diffusion metrics. For instance, most commercial software used in clinical practice does not allow pre-processing of DWI by image registration and noise filtration, which can significantly affect quantitative measurements. In addition, post-processed DWI sequences might cause an overlap between the ADC values of malignant and non-malignant tissues (72).

#### 3.1.3 Perfusion-weighted imaging

Perfusion refers to the delivery of blood to the end organ at the level of the capillaries. PWI is a non-invasive MRI tool capable of measuring cerebral perfusion using specific hemodynamic parameters. Three types of PWI approaches have been developed to acquire this information using both plain and contrast-enhanced sequences. Dynamic susceptibility contrast (DSC-MRI) and dynamic contrast enhanced (DCE-MRI) are the two types that use contrast agents, while arterial spin-labeling (ASL) does not require administration of exogenous contrast agents as it uses blood as an endogenous tracer (73).

DSC-MRI is more specifically used in brain imaging, unlike the other two types. This technique involves the rapid intravenous injection of a bolus of a paramagnetic contrast agent while obtaining a serial measurement of the signal change of the T2- or T2*-weighted MRI. Subsequently, concentration time curves are obtained that lead to the calculation of quantitative maps that depict cerebrovascular hemodynamic parameters such as cerebral blood volume and flow rate. Low spatial resolution and signal loss artifacts due to the metallic surgical implants and other abnormalities such as calcification and dense bones are several disadvantages associated with DSC-MRI (74).

DCE-MRI is a standardized PWI technique that requires the administration of a contrast agent; T1-weighted MRI images are acquired dynamically before, during, and after the injection of the bolus of contrast agent. The information obtained is interpreted as permeability characteristics of the tissues based on tracer kinetic modeling principles. These extracted features from regions of DCE-MRI, such as *K_trans_*, *K_ep_*, *V_e_*, and *V_p_*, can describe the vascular micro-environment, including angiogenesis in brain tumors. Angiogenesis plays a pivotal role in the growth of sporadic VS, and that has been the reason for using DCE-MRI in two of the three included VS studies (61, 62). In high-flow lesions, including meningiomas, the kinetic parameter *K_trans_* is permeability-limited (28). Therefore, several included studies used DCE-MRI kinetic parameters to analyze meningioma molecular markers (22, 25, 28).

ASL is a PWI technique that uses magnetically labeled arterial blood as an endogenous diffusible tracer to measure cerebral blood flow. Thus, ASL is recognized as a completely noninvasive and safe imaging tool that does not require the administration of contrast agents and can be repeated for frequent assessments. This imaging technique has limitations related to methodological shortcomings and artifacts when imaging the posterior fossa (75).

#### 3.1.4 Positron emission tomography

PET is an imaging tool where *in vivo* biochemical and physiological processes, such as metabolism and blood flow, are visualized using radioactive substances known as PET tracers, providing unique functional information about the tumor (76). PET tracers have been used on specific molecular targets during the past few decades, but few have been demonstrated to be clinically relevant. PET tracer traditionally used in tumor imaging is 18F-2-fluoro-2-deoxy-D-glucose (18F-FDG). This tracer is use to distinguish recurrent tumors from radiation necrosis (77). It is a glucose analog that is actively transported into the metabolically active cells, phosphorylated, and trapped intracellularly. Malignant cells have an increased energy demand, resulting in high glucose consumption and an upregulation of glucose transport compared to other cells, resulting in increased accumulation of FDG (78). However, FDG has shown limitations in brain tumor imaging due to the high glucose consumption of the surrounding healthy brain parenchyma, thus decreasing PET imaging sensitivity (78). Another known PET tracer for brain tumor imaging is a nucleoside analog called 3’-deoxy-3’-fluorothymidine (18F-FLT) (77). This tracer can limit the uptake of 18F-FLT by healthy brain tissues. Several included studies used 18F-FLT to find associations with the progression of tumors. In meningiomas, a correlation was found between the uptake of 18F-FLT and the Ki-67 molecular marker, in addition to the association with the progression of the tumor reported by Bashir et al. (33); Bashir et al. (29).

Consequently, amino acid PET tracers, such as 11C-methyl-L-methionine (11C-MET), O-(2-[18F]fluoroethyl)-L-tyrosine (18F-FET), and 3,4-dihydroxy-6-[18F]-fluoro-L-phenylalanine (18F-FDOPA), have been used due to their high uptake in neoplastic tissue and relatively low uptake in healthy brain tissues (78). Amino acid PET has been used in several scenarios, including the detection and precise delineation of neoplastic tissue when conventional MRI is inconclusive and the determination of the post-radiation treatment effects that yield progression and/or recurrence. Since meningiomas have a strong expression of somatostatin receptor subtype 2, PET with somatostatin receptor ligands (68Ga-DOTATOC, 68Ga-DOTATATE) is used (78). In a few studies, the uptake of 68Ga-DOTATOC was found to be related to treatment outcomes and the VEGF molecular marker in meningiomas (24, 34). This PET is reported to be useful for differentiating the normal pituitary tissue from the pituitary adenomas (79).

Standard uptake value (*SUV*) is a common metric taken from PET imaging that depicts a relative measure of radiotracer uptake (80). Other metrics, such as the tumor-to-blood ratio (TBR), that correlate to the metabolic rate of the radiotracer, are used to overcome shortcomings such as time dependence and susceptibility to errors caused by dose calibration and the scanner in the *SUV* metric.

SPECT is a similar nuclear imaging technique to PET, but it is less expensive and uses radiotracers. SPECT measures gamma-rays, whereas PET uses positrons to measure the decay of the specific radiotracers. PET is considered a more sensitive nuclear imaging technique than SPECT (81).

### 3.2 Imaging biomarkers of different tumors

In this section, we discuss imaging biomarkers we identified through this systematic review, categorized based on tumor neoplasm.

#### 3.2.1 Meningiomas

##### 3.2.1.1 Imaging biomarkers associated with molecular and histopathological markers

VEGF is a histopathological marker that correlates with tumor vascularity, vascular permeability, malignancy, progression-free survival, and overall survival of meningiomas (82–84). Hence, non-invasive imaging tools such as SPECT, DSC-MRI, and DCE-MRI have been used to find imaging biomarkers associated with the VEGF marker. Takeda et al. (21) identified significant differences in the Thallium-201 (Tl) uptake index of Thallium-201 chloride single-photon emission CT (Tl SPECT) between VEGF weakly and strongly positive tumors. In their study, they calculated the Tl uptake index by dividing the mean value obtained from the tumor region by the mean value extracted from the non-tumor region. Similarly, the association between the VEGF biomarker and a cerebral blood volume (CBV) marker extracted from dynamic susceptibility-weighted contrast-enhanced perfusion MRI (DSC-MRI) was assessed for meningiomas by Ginat et al. (22). This study extracted the maximum CBV manually from the tumor region, excluding areas containing necrosis, cysts, hemorrhage, large vessels, or calcification. A relative CBV (rCBV) value was computed as a ratio between the intratumoral maximum CBV value and contralateral cerebral white matter CBV, which provides the highest inter-/intra-observer reproducibility (85). They observed a significantly positive correlation between rCBV and VEGF scores. Keil et al. (28) assessed the ability to use the DCE-MRI kinetic parameters for predicting the VEGF marker *via* linear regression analysis. However, their results did not demonstrate a reliable prediction of VEGF, concluding that the DCE-MRI-derived kinetic parameters may not be able to be used as an imaging biomarker for meningioma. In recent studies, research has focused on finding associations with the PET-related metrics, and Bashir et al. (34) demonstrated that the [68Ga]Ga-DOTA-TOC PET metrics, the *SUV_mean_* and *SUV_max_*, all positively correlate with VEGF in meningiomas.

Ki-67/MIB-1 labeling index is a biomarker used to distinguish proliferating and quiescent cells, with an elevated Ki-67 index typically associated with a less favorable clinical outcome in many tumors (86, 87). Tang et al. (23) used ADC values extracted from DWI to find a correlation with the Ki-67 proliferation index in meningiomas. Regions of interest were annotated on the ADC maps, excluding the cystic and necrotic areas, which were identified using conventional MRI, and then the mean ADC values were extracted. The observations suggest that the ADC value inversely correlates with the Ki-67 index and, thus, can be used to differentiate the aggressiveness of meningiomas. Later, this was further proved by the work done by Lu et al. (32). Bozdağ et al. (39) also demonstrated the negative correlation between ADC and the Ki-67, additionally stating that meningiomas with necrosis have a lower ADC compared to non-nectrotic meningiomas. However, Lu et al. (32) found a positive correlation between ADC extracted from the edema region and Ki-67. Moreover, Gihr et al. (26) have assessed the correlation between the additional parameters extracted from the ADC histogram profile and the Ki-67. A positive correlation is identified between the entropy and the Ki-67, revealing the entropy as a promising imaging biomarker for presurgical grading. Takeda et al. (21) recognized a correlation between the delayed Tl uptake index and the MIB-1 labeling index with *p<*0.0001. In addition to these imaging tools, PET imaging has been used to find a relation to the Ki-67 proliferation index. Bashir et al. (33) have identified a correlation with the 3’-deoxy-3’-[18F]fluorothymidine (18F-FLT) PET/MRI metrics, *SUV_max_* and *SUV_mean_*.

Microvessel density (MVD) is a surrogate marker used to measure the angiogenesis and blood vessel formation of tumors. Due to the rapid growth of malignant tumors, microvessel formation is relatively low due to ischemia and hypoxia. Hence, an association between MVD and prognosis has been analyzed in many studies for different intracranial tumor types, including meningiomas, gliomas, and pituitary tumors (88, 89). Jensen and Lee (84) did not observe any statistical difference in MVD between high- and low-grade meningiomas. However, contrary to this study, Shi et al. (25) showed a significantly higher MVD value in benign meningiomas compared to malignant meningiomas. Additionally, they assessed the association of various PWI parameters with MVD in meningiomas, demonstrating a statistically significant positive correlation between rCBV and MVD.

Fibrotic tumor vessels (FTV) are another marker related to the vessel environment and were identified to have associations with the recurrence of tumors, vessel density, and VEGF in a study conducted by Hess et al. (35). They further recognized FTV to have associations with morphological characteristics on T1 post-contrast MRI, disruption of the arachnoid layer, and irregular shape in tumors, speculating that these imaging biomarkers might serve as predictors of underlying histopatological markers of meningiomas.

##### 3.2.1.2 Imaging biomarkers associated with meningioma grades

In addition to finding associations between imaging markers and the different invasive histopathological or gene markers such as Ki-67 and VEGF, in the past decade research has been conducted to find the association of imaging markers with different meningioma grades, reflecting meningioma prognosis. Gihr et al. (26) used histogram profiling of ADC maps to distinguish low- and high-grade meningiomas. In this study, they obtained the following set of first and second order features: mean ADC, max ADC, min ADC, percentile 10, 25, 75, and 90 ADC, median ADC, skewness, kurtosis, and entropy, from the histogram profile of the ADC map of the whole tumor. The results demonstrate that the percentile, mean, and median ADC values are significantly lower in high-grade meningiomas compared to those in the low-grade group. This observation was further proved in later studies (38, 39). However, the entropy was significantly higher in high-grade meningiomas compared to low-grade meningiomas. More recently, Buizza et al. (40) demonstrated that several other features extracted from DWI, such as median ADC, water intrinsic diffusivity and radius, cell volume fraction, and apparent cellularity, are significantly different between high-grade (WHO grades 2 and 3) and low-grade (WHO grade 1) meningiomas.

Later, Gihr et al. (27) extended their initial study (26) on meningiomas to assess the ability to use post-contrast T1 instead of DWI. They did not observe any significant difference in first order characteristics between low and high grade meningiomas, according to previous studies. However, they did observe a subtle difference in second-order characteristics, such as entropy and skewness, between both groups and suggested future research with a larger patient cohort to achieve statistical significance. Park et al. (36) assessed features that might explain complexity of structures to predict meningioma grades using post-contrast T1. They demonstrate that the fractal dimension may be used as an imaging biomarker to predict the grade of meningiomas. Sun et al. (37) analyzed tumor location on post-contrast T1 MRI to differentiate the biological characteristics of meningiomas. Their observations indicate that the grade 2 and 3 meningiomas present a strong predominance in the frontal structures compared to the grade 1 meningiomas. Subsequently, Yu et al. (38) also assessed conventional T1 and T2 characteristics for different meningioma grades. They observed that WHO grade 3 tumors have a large maximum tumor diameter and a high area of peritumoral edema compared to the lower grades (1 and 2). In addition, the enhancement degree and patterns (homogeneous or heterogeneous), lobulation (shape of the tumor), flowing voids (blood flow as a signal on MRI), and dural tail (indicating the thickening of the dura adjacent to the tumor) were significantly different between any two grades. In contrast to this study, Bozdağ et al. (39) found no significant difference between the presence of peritumoral edema on conventional MRI in low- and high-grade meningiomas. Additionally, they also observed no significant difference in the irregularity of the tumor margin and the presence of bone invasion.

Recently, a machine-learning-based study has used imaging features to classify meningioma grades. Chen et al. (30) extracted texture features from post-contrast T1.

##### 3.2.1.3 Imaging biomarkers associated with clinical outcomes

Apart from assessing the grade of meningiomas, some studies have also considered clinical outcomes such as complications, operative time, tumor recurrence, and functional status [using the Karnofsky Performance Status scoring system (90)] to develop or identify imaging biomarkers. Loewenstern et al. (31) evaluated the relationship between peritumoral edema and clinical outcomes quantitatively using conventional MRI, T1, and T2 MRI. They obtained a measurement called the Edema Index, by dividing the peritumoral edema volume by the whole tumor volume. This index shows an association with functional decline after surgery in older patients. This research group has extended this work by assessing the association between the Edema Index and mutational burden (42), observing that tumor edema is associated with brain invasion and reduced overall survival. Subsequently, Bashir et al. (29) used TBR metrics from the (18F-FLT) PET, observing increased (18F-FLT) uptake in progressive asymptotic meningiomas.

Clinical outcomes of treatments such as proton therapy have also been examined in the past few years. Buizza et al. (40) utilized DWI to recover markers of tumor microstructure by longitudinal analysis pre- and post-treatment. The increment in the values for median ADC, water intrinsic diffusivity, and radius and the decrement in the values for cell volume fraction and apparent cellularity are observed in the post-treatment DWI for high-risk meningiomas. Feraco et al. (41) also conducted similar research, where they used the relative ADC mean (rADCm) to assess longitudinal volume changes. Their results indicated a statistically significant difference in rADCm between pre- and post-proton therapy treatment, with significant, progressively increasing rADCm values at each time point. Subjects that showed 20% or more volume reduction after treatment had significantly lower pre-treatment rADCm values.

Seystahl et al. (24) conducted a study to find the outcomes of the somatostatin-receptor (SSTR)-targeted radionuclide therapy treatment for meningiomas using the 68Ga-DOTATOC PET/CT. The results demonstrate that the *SUV_mean_* and *SUV_max_* is significantly low in WHO grade 2 tumors, which had shown progression after 6 months of the treatment. The multivariate regression analysis has shown the high grade and the low *SUV_mean_* are associated with the progression at 6 months, and higher uptake is associated with longer progression-free survival.

#### 3.2.2 Pituitary tumors

##### 3.2.2.1 Imaging biomarkers associated with molecular and histopathological markers

Similar to the case of meningiomas described in the previous section, biomarkers such as VEGF, MVD, and Ki-67/MIB-1 may also depict pituitary tumor progression and outcomes (88). In an earlier study, Pan et al. (43) performed an analysis in which a significantly higher Ki-67 was observed in the presence of invasion on post-contrast T1 MRI compared to non-invasive pituitary adenomas. Similar observations have been made in other studies (53), with a higher Ki-67 index seen in invasive pituitary adenomas. In recent studies, more imaging tools have been used to investigate associations with the Ki-67 index. Conficoni et al. (55) utilized conventional MRI and DWI to predict the Ki-67 index. They observed a negative correlation between the enhancement ratio, the ratio between the signal intensity in post-contrast T1 and pre-contrast T1 within the solid region of the tumor, and the Ki-67 index. Nonetheless, the mean ADC value showed a negative correlation with the Ki-67 index. In other recent studies, the Ki-67 labeling index was predicted using 1,128 quantitative imaging features extracted from preoperative T2-weighted MRI (54). These features include both first-order histograms and high-order textural features, with and without various filters such as wavelets to derive hidden textural features. However, Mahmoud et al. (44) have not found a significant correlation between the ADC values and the MIB-1 labeling index in pituitary adenomas; Tamrazi et al. (48) have determined an inverse correlation between mean ADC values and the MIB-1 labeling index in pituitary macroadenomas.

Pan et al. (43) also demonstrated higher MVD present in invasive adenomas compared to non-invasive adenomas, which has been confirmed later in other published research (45). Studies also reveal that the invasion of adenomas is associated with VEGF expression, another marker of less favorable outcomes for tumors (43).

##### 3.2.2.2 Imaging biomarkers associated with functioning/non-functioning pituitary adenomas

Pituitary adenomas are also categorized based on various hormone secretory functions. Mahmoud et al. (44) used conventional T1 and T2 MRI along with DWI to differentiate these different tumor categories. They observed a significantly lower mean and minimum signal intensity on T2-weighted MRI for growth hormone-secreting adenomas compared to others. Park et al. (51) demonstrated significantly high ratios of tumor width/anteroposterior diameter on conventional MRI in non-functioning adenomas with hyperprolactinemia. These hormone-secreting pituitary adenomas are typically considered benign based on histology, but there is an underlying significant morbidity due to direct mass effects such as defects in visual fields and/or hyper-secretion of hormones, which results in a shortened lifespan (91, 92).

According to the literature, sparsely granulated adenomas are comparatively more aggressive, and therefore imaging biomarkers related to granulation have also been analyzed in the past decade. A higher T2 intensity was identified in sparsely granulated adenomas compared to densely or intermediately granulated adenomas (46). This observation has been confirmed in more recent studies (57, 58). Swanson et al. (57) also demonstrated size increment and invasive behavior in sparsely granulated adenomas. Park et al. (56) recently developed a machine-learning-based model to predict the granulation pattern in growth hormone-secreting pituitary adenomas using shape and first- and second-order features extracted from the post-contrast T1 and T2 weighted MRI.

##### 3.2.2.3 Imaging biomarkers associated with clinical outcomes

Simultaneously, research has been conducted to find the imaging biomarkers that can correlate with treatment responses, recurrence, and outcomes. Heck et al. (46) have reported homogeneity within the adenoma on the T2 MRI as a marker of tumor size reduction after the somatostatin analog treatment. Galm et al. (50) have extracted textural features, namely the mean, median, maximum, and minimum intensities of the tumor region; skewness; measure of asymmetry of the intensity distribution; kurtosis; and degree of peaking in the intensity distribution, from the T1-weighted MRI. Cox proportional hazards regression analysis subsequently showed that the mean, median, minimum, and maximum pixel values of pituitary adenomas were all associated with recurrence and progression following surgery. Fan et al. (52) have used T1, T2, and post-contrast T1-weighted MRI to predict the responses to radiotherapeutic treatments for acromegaly patients. They extracted 1,561 imaging features from the tumor region, including first-order, textural, wavelet features, size, and shape features. The final radiomic signature developed for response prediction includes one shape, two textural, and three wavelet features, selected using the leave-one-out cross-validation technique. In another recent study by Zhang et al. (59), the same radiomic features were extracted from post-contrast T1 MRI and machine learning was used to predict the recurrence of pituitary macroadenoma within 5 years. They concluded that the combination of clinicopathological features and images is useful for recurrence prediction and is superior to prediction using only clinical features.

Ceccato et al. (47) observed that radiological invasion is typically present in aggressive pituitary adenomas. Hasanov et al. (53) demonstrated that invasion of the cavernous sinus is associated with recurrence. Thus, these studies verify that tumor invasion can be considered an imaging biomarker in pituitary tumors related to prognosis. Some studies have also searched for other imaging biomarkers associated with the invasiveness characteristic of pituitary tumors. Alhambra-Expósito et al. (49) also demonstrated that hyperintense adenomas are more invasive than hypointense adenomas.

#### 3.2.3 Vestibular schwannoma

##### 3.2.3.1 Imaging biomarkers associated with molecular and histopathological markers

The study of imaging biomarkers in VS has included the evaluation of biological processes such as cell proliferation and vessel density, including Ki-67 and microvessel density markers. de Vries et al. (60) obtained size measurements (the largest tumor diameter), an evaluation of tumor density (homogeneous, inhomogeneous, and cystic), and a tumor growth index (maximal tumor diameter/age of the patient) using post-contrast T1 and T2 images. They reported no relation between these features and the Ki-67 index. In their results, MVD shows a significantly positive correlation with tumor size and tumor growth index.

##### 3.2.3.2 Imaging biomarkers associated with tumor growth

Some studies identified imaging biomarkers associated with tumor growth. Lewis et al. (61) utilized both PET with the 11C-(R)-PK11195 tracer and DCE-MRI to investigate the relationship between inflammation and tumor growth in sporadic VS. The results demonstrated the binding potential of 11C-(R)-PK11195, and that values were significantly higher in growing tumors relative to static ones. In another study, Lewis et al. (62) assessed the relationship between diffusion metrics (e.g., mean diffusivity and fractional anisotropy) extracted from the DCE-MRI and tumor growth rates in both NF2 and sporadic VS. They demonstrated that and tissue extravascular–extracellular space *v_e_*, increased with the increasing tumor size in both types.

#### 3.2.4 Solitary fibrous tumors

The identification of imaging biomarkers has been conducted, focusing on the phenotypes and the grading of SFT. Grade 2 and 3 SFTs are classified based on mitotic activity, and thus, the studies have been conducted to predict the grade before surgery using imaging biomarkers. Therefore, the imaging features associated with the Ki-67 index have been assessed in several studies. Lu et al. (32) identified a statistically significant negative correlation between the ADC of the lesion and the Ki-67 in grade 2 SFT. This was also later observed by Li et al. (64). Moreover, Lu et al. (32) found a significantly positive correlation between ADC extracted from the edema region and Ki-67. These observations were on par with their observations of meningiomas in the same study.

Mama et al. (63) have identified imaging features related to the HPC phenotype using conventional MRI and ADC maps. They observed that the grade 2 HPCs had higher ADC values, whereas the grade 3 values (which were more aggressive and malignant than the grade 2 HPCs) were slightly lower. Li et al. (64) also verified these observations, with significantly different ADC values between grade 2 and 3 SFTs.

## 4 Discussion

### 4.1 Critical assessment of the included studies

There were certain biases in patient selection in the included studies. Most of the studies used relatively small datasets, usually because of the limited availability of clinical data, likely resulting in selection bias (29, 30). Consequently, the sample populations and the target populations varied significantly, which may limit the ability to generalize the observations and findings from these studies. For example, Ugga et al. (54) excluded patients with extensively necrotic and hemorrhagic lesions from the study. Furthermore, 75% of the included studies did not clearly mention if patients were selected consecutively. Approximately 12% of the studies were considered to have a ‘high’ risk of bias as they did not mention the time period in which patients were enrolled, the exclusion criteria, or whether a consecutive or random sample was used. Approximately 14% of the included studies clearly mentioned all the above factors and satisfied the criteria. Those were considered to have a ‘low’ risk of bias in patient selection.

Hasanov et al. (53) extracted tumor size from the MRIs but did not clearly mention the feature extraction process or whether it was done automatically or performed manually by an expert. This made it unclear whether the index test had caused a risk of bias. Yu et al. (38) extracted MRI characteristics to find associations with the WHO grades of meningiomas. However, they did not mention if the feature extraction and the labeling of WHO grades were done by independent experts, which made the risk of bias unclear. Similarly, 50% of the included studies did not mention the independent and blinded extraction of the features, i.e., the index test, and were thus considered to have an ‘unclear’ risk of bias. Ceccato et al. (47) mentioned that they used radiological images but did not specify which imaging type was used and were thus excluded, leading to reporting biases in the index test. Lewis et al. (61) clearly mentioned that their study was unblinded. Therefore, both of those studies (6.8% of the included studies) were considered to have a ‘high’ risk of bias in the index test. Approximately 43% of the included studies interpreted the index test results without knowledge of the results of the reference standard.

In this review, we considered several types of adverse outcome-related factors, such as molecular and histopathological markers, progression, invasiveness, recurrence, and grading of tumors. For these different outcomes, the studies used appropriate reference standards to categorize the patients. Bashir et al. (29) used the trial end-point criteria from the Response Assessment of Neuro-Oncology (RANO) workgroup (93) and considered the tumor to be progressing when there is a 25% increment in the product of two maximal perpendicular diameters (2D) of the tumor in comparison to the baseline. Therefore, the standard reference interprets the target condition, i.e., progression, appropriately. Similarly, appropriate and standardized reference standards were used in 38% of the included studies, which were interpreted without the knowledge of the index test.

The concerns regarding applicability were low, with almost all the included studies aligning with the review question we address.

**Figure 2:**
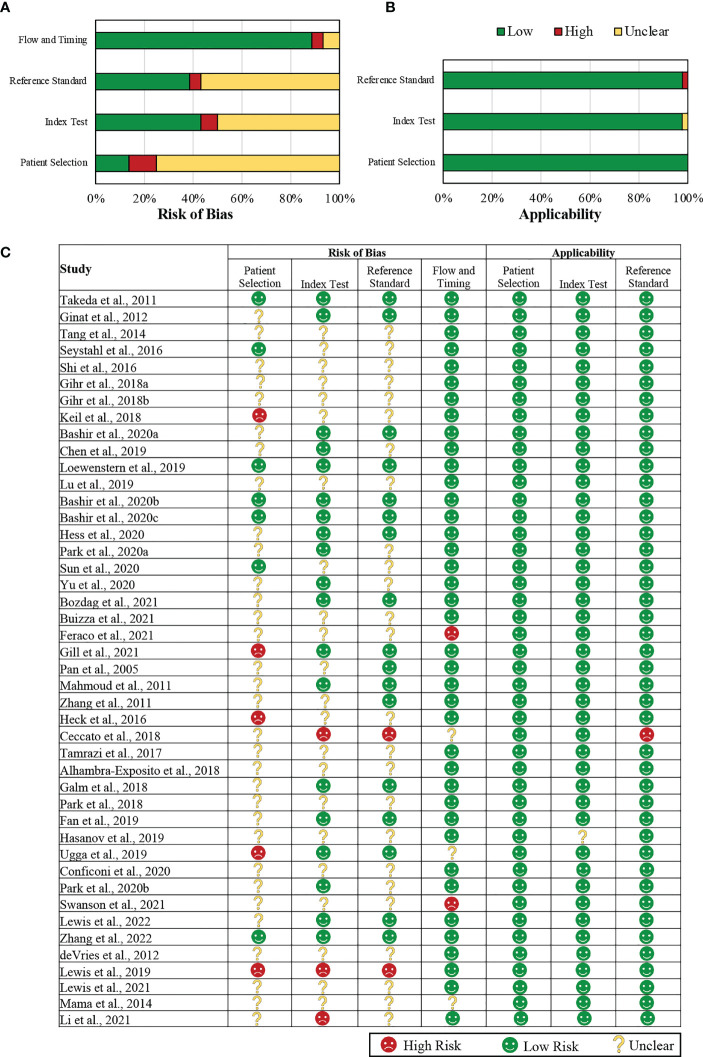
Summary of the QUADAS-2 assessments of the included studies. **(A)** Graphical representation of included studies (in percentages) in each key domain in terms of the risk of bias. **(B)** A graphical representation of the included studies (in percentages) in each key domain in terms of the concerns regarding their applicability. **(C)** A tabular representation of the assessments assigned for each included study. QUADAS-2: Quality Assessment of Diagnostic Accuracy Studies-2.

Details of the QUADAS-2 assessment are summarized in **Figure 2**.

### 4.2 Overall assessment

In this systematic review, we identified studies that investigated imaging biomarkers of extra-axial intracranial tumors. Included studies predominantly focused on the association and correlation of imaging biomarkers with tumor growth. Others relate to the association of imaging biomarkers with molecular or histopathological tumor markers.

With the advancement of high-throughput technologies during the past decade, research was conducted to find the molecular markers of all types of tumors. Acquisition of molecular markers requires biological samples obtained using an invasive approach (biopsy or surgery). Surgical biopsy always provides the most definitive means of diagnosis, but it is associated with surgical risk and additional costs. Heterogeneity within the tumor also means that different areas may yield different molecular results. However, for diagnosis and screening, imaging tests such as MRI that are already obtained as part of the routine clinical workflow present an opportunity to recognize underlying molecular markers without the need for an invasive biopsy. Moreover, imaging biomarkers can also overcome the intra-tumor heterogeneity, providing consistent predictions. This can lead clinicians to take critical decisions at the right time, ultimately optimizing the personalized management of tumors.

For meningiomas, the majority of studies assessed imaging biomarkers that depict the underlying molecular or histopathological biomarkers such as MVD, Ki-67 index, and VEGF. Since the WHO has given a grading system based on aggressiveness and histopathology, several other included studies have assessed imaging biomarkers that relate to the grade or aggressiveness of the meningioma.

In addition, we observed PET imaging metrics showing relationships to different underlying molecular markers with different PET tracers for meningiomas. Bashir et al. (33) demonstrated a correlation between Ki-67 and 18F-FLT PET/MRI metrics, while in another study by Bashir et al. (34), where he used the [68Ga]Ga-DOTA-TOC PET tracer, no correlation with Ki-67 was found. Furthermore, in Bashir et al. (34), they found a correlation between [68Ga]Ga-DOTA-TOC PET metrics and VEGF, but this was not observed with the 18F-FLT PET tracer.

For pituitary tumors, most studies focused on the correlation between the tumor invasion of surrounding structures and the underlying histopathology. In particular, aggressiveness is often correlated to how invasive the lesion is found to be, either intra-operatively or on diagnostic imaging (47). The fourth edition of the World Health Organization (WHO) classification of pituitary tumors recommends evaluation of tumor proliferation and invasion to identify aggressiveness (94). Zhang et al. (45) further distinguish the invasive adenomas as having significantly greater tumor diameters and volumes. In pituitary adenomas (PA), invasiveness has been shown to be the main contributing factor to recurrence and poor prognosis (95). Most of the early studies focused on using the invasiveness of the lesion as an imaging marker for prognosis; however, subsequent to this, other imaging biomarkers that can be extracted from more modern imaging techniques were assessed with increasing interest.

An imaging-based grading system based on the invasion of pituitary tumors was proposed by Knosp et al. (96). In this grading system, grades 0 and 1 mean no invasion, grade 2 is assigned when there is a probable invasion, and grades 3 and 4 indicate a cavernous sinus invasion. The majority of studies that assessed invasion as an imaging biomarker used this grading system (43, 58). As well as the Knosp system, a scoring system proposed by Cottier et al. (97) has also been used in a few studies (45). This scoring system assesses the percentage of the intra-cavernous internal carotid artery encased by the adenoma.

For VS, there were a very limited number of studies that assessed imaging biomarkers. Conventional MRI was used only in a single study where they found an association with histopathological markers of VS (60). Limited availability of patient cases with serial MRI scans restricted them from analyzing imaging biomarkers associated with the tumor growth in depth.

To clearly distinguish between two and three SFTs, surgery is necessary. Since both of these types are also malignant, research has been conducted to identify the tumor grade using pre-operative medical images, which can allow clinicians to formulate personalized treatment plans. However, the number of patients used in all the included studies on SFTs is limited due to the low incidence rate.

### 4.3 Future directions

Considering the included extra-axial brain tumor studies, the majority of the studies extracted features by determining the region of interest manually (54, 59). This is a time-consuming task that requires clinical experts. Future work can focus on automating the segmentation task using deep learning. This will lead to more deep feature extraction and analysis. Moreover, automated feature extraction, unlike manual feature extraction, is likely to result in reduced inter-observer variability. In the future, such techniques may be adapted to analyze the growth or progression of extra-axial tumors too. This has the potential for more personalized and standardized management of extra-axial tumors. To assess the impact of such automated methods, it would be worthwhile to test their use in simulated clinical workflows before assessing their effectiveness in the clinic.

### 4.4 Limitations

As we recognized through this review, the major limitation is the limited usage of machine learning, and in particular deep learning. The major reason behind this may be the lack of large-scale annotated datasets. Most of the included studies used private single-institutional datasets (27, 30; 612 59). These datasets could be made public for common use. This might lead to better reproducible and transparent research. Further, multi-institutional datasets will produce more persistent results.

The present systematic review was limited by various factors. Firstly, given the variety of ways data were presented and the relatively small number of available studies, it was not possible to perform a meta-analysis and quantitatively analyze the data. Consequently, we could not draw any firm conclusions concerning the effectiveness of the described imaging techniques and biomarkers. Secondly, the studies were of mixed methodological quality, reporting a variety of imaging biomarkers, limiting our discussion to qualitative and narrative discussion.

## 5 Conclusions

A limited number of studies have assessed imaging biomarkers related to intracranial extra-axial tumors. Future work should focus on using serial images and longitudinal patient data to develop composite imaging and clinical imaging biomarkers capable of predicting tumor behavior and growth. Such work would be particularly beneficial for the management of extra-axial tumors, pathologies that are typically benign and where surveillance management is commonly employed. This review provides a guide to the features researchers can utilize for developing reproducible and standardized imaging biomarkers.

## Data availability statement

The original contributions presented in the study are included in the article/supplementary material. Further inquiries can be directed to the corresponding author.

## Author contributions

NW conceived the manuscript. NW and OM performed the systematic literature search. NW drafted the initial manuscript. JS and TV reviewed the final manuscript. All authors contributed to the article and approved the submitted version.

## Footnotes

1
https://www.crd.york.ac.uk/prospero.

2
https://www.covidence.org.
